# Vitamin D and Endometrium: A Systematic Review of a Neglected Area of Research

**DOI:** 10.3390/ijms19082320

**Published:** 2018-08-08

**Authors:** Greta Chiara Cermisoni, Alessandra Alteri, Laura Corti, Elisa Rabellotti, Enrico Papaleo, Paola Viganò, Ana Maria Sanchez

**Affiliations:** 1 Reproductive Sciences Laboratory, Division of Genetics and Cell Biology, IRCCS San Raffaele Scientific Institute, 20132 Milano, Italy; cermisoni.gretachiara@hsr.it (G.C.C.); vigano.paola@hsr.it (P.V.); 2 Obstetrics and Gynaecology Department, IRCCS San Raffaele Scientific Institute, 20132 Milano, Italy; alteri.alessandra@hsr.it (A.A.); corti.laura@hsr.it (L.C.); rabellotti.elisa@hsr.it (E.R.); papapelo.enrico@hsr.it (E.P.)

**Keywords:** Vitamin D, endometrium, endometrial cancer

## Abstract

Growing evidence supports a role of vitamin D (VD) in reproductive health. Vitamin D receptor (VDR) is expressed in the ovary, endometrium, and myometrium. The biological actions of VD in fertility and reproductive tissues have been investigated but mainly using animal models. Conversely, the molecular data addressing the mechanisms underlying VD action in the physiologic endometrium and in endometrial pathologies are still scant. Levels of VDR expression according to the menstrual cycle are yet to be definitively clarified, possibly being lower in the proliferative compared to the secretory phase and in mid-secretory compared to early secretory phase. Endometrial tissue also expresses the enzymes involved in the metabolism of VD. The potential anti-proliferative and anti-inflammatory effects of VD for the treatment of endometriosis have been investigated in recent years. Treatment of ectopic endometrial cells with 1,25(OH)_2_D_3_ could significantly reduce cytokine-mediated inflammatory responses. An alteration of VD metabolism in terms of increased 24-hydroxylase mRNA and protein expression has been demonstrated in endometrial cancer, albeit not consistently. The effect of the active form of the vitamin as an anti-proliferative, pro-apoptotic, anti-inflammatory, and differentiation-inducing agent has been demonstrated in various endometrial cancer cell lines.

## 1. Introduction: Vitamin D, Metabolism, and Reproduction

Vitamin D is a well-known steroid hormone whose activated form is the result of the conversion of 7-dehydrocholesterol in the skin, under the influence of ultraviolet B light. To become active, it requires two hydroxylation steps: a 25-hydroxylation occurring mainly in the liver, leading to 25-hydroxyvitamin D_3_ (25-OHD_3_), and a 1α-hydroxylation occurring in the proximal tubules cells of the kidney, leading to 1,25-dihydroxyvitamin D_3_ (1,25(OH)_2_D_3_). All these hydroxylation steps are catalyzed by cytochrome P450 mixed-function oxidases that are produced by the CYP gene superfamily group. These enzymes are located in the liver either in the endoplasmic reticulum (e.g., CYP2R1/25-hydroxylase) or in the mitochondria (e.g., CYP27A1/25-hydroxylase) or in the kidney mitochondria (e.g., CYP27B1/1α-hydroxylase) [1]. In terms of catabolism, CYP24A1/24-hydroxylase catalyzes the conversion of both 25-OHD_3_ and 1,25(OH)_2_D_3_ into a series of 24- and 23-hydroxylated products targeted for excretion along well-established pathways concluding in the water-soluble biliary metabolite calcitroic acid [2]. However, several extrarenal tissues, such as bone, bone marrow, prostate, and macrophages, also express actively the 1α-hydroxylase enzyme [3].

The biological actions of vitamin D (VD) are mainly mediated by VD receptor (*VDR*) that belongs to the nuclear receptor superfamily. Upon 1,25(OH)_2_D_3_ binding, VDR forms a heterodimer complex with the retinoid X receptor (*RXR*) and interacts with regions of the DNA named vitamin D response elements (*VDRE*). These elements recruit coactivators able to regulate the transcription of target genes involved not only in calcium and phosphate homeostasis [4] but also in cell proliferation, differentiation and immune response [5,6]. Moreover, some evidence suggests that non-genomic pathways mediate rapid signaling though cytosolic and membrane VDR [7].

VDR is expressed in different organs and tissues including skeleton, immune system, parathyroid glands and reproductive tissues [8]. Several studies have demonstrated that the ovary could be a target organ for 1,25(OH)_2_D_3_ raising the possibility that this active metabolite plays a role in modifying ovarian activity [9]. The role in fertility and reproductive capacity has been investigated in animal models for many years, demonstrating that 25-OHD_3_-deficient female rats had reduced fertility rates [10]. VDR knockout female mice are unable to reproduce due to defects in uterine development [11]. Furthermore, 1α-hydroxylase-null mice manifest a decrease in ovarian size and uterine hypoplasia [12]. Finally yet importantly, the role of VD in uterine physiology seems to be essential for the normal differentiation of decidual cells [11,13]. 

Beyond the physiology, there is several evidence on the possible effect of VD in endometrial pathology. For example, the role of VD has been studied in endometriosis and endometrial cancer.

Endometriosis refers to the presence of functionally active endometrial tissue, glands, and stroma in ectopic sites. Its real prevalence in the female population is unknown mainly due to overlooked and delayed diagnosis but it has been reported to affect about 5–10% of women of reproductive age [14]. Several theories have been proposed for its etiopathogenesis with the retrograde menstruation hypothesis being the most accepted [15]. However, this hypothesis has many limitations, which numerous other theories have attempted to circumvent [16,17], including altered Genetic/Epigenetic-based mechanisms [18].

The endometrium of women with endometriosis presents abnormalities on a structural as well as on a functional level affecting proliferation ability, presence of immune components, adhesion molecule expression, steroid and cytokine productions, compared with the endometrium of women without the diseases [19,20]. Moreover, endometriosis possesses features similar to a malignancy and fulfills several criteria of an autoimmune disease. VD is a known anti-proliferative, anti-inflammatory, and even an immunomodulatory agent. Therefore, the association between endometriosis and VD has been the object of some investigation [21]. Unfortunately, data linking VD action and endometriosis on a molecular level is still sparse, both in terms of a potential role in the pathogenesis and therapeutics.

Endometrial cancer is the most common female gynecological malignant pathology in developed countries and its incidence is increasing [22]. A recent systematic review of literature found out a crucial role of VD in cancers as high circulating levels of VD are associated with a reduced risk of developing certain cancer types (breast, colorectal, gastric, hematological, head and neck, kidney, lung, ovarian, pancreatic liver, prostate, and skin cancer) [23]. Nevertheless, the association between VD and endometrial cancer risk is at present controversial [24,25,26]. Therefore, a better investigation of the potential molecular mechanisms at the basis of the local action of VD in endometrial cancer could be of a value.

Encouraged by the aforementioned information, we aimed to present a systematic review on all available molecular data related to the effect of VD in human endometrium and endometrial diseases, with a focus on endometriosis and endometrial cancer.

## 2. Methods

The search strategy was agreed upon a priori by the authors. We searched in PubMed Database for articles published in the English language using the following MeSH search terms: ‘’vitamin D” AND “endometr*” with restriction to the human species. We included articles referring to physiological endometrium, endometriosis, and endometrial cancer with available molecular data. 

**Figure 1:**
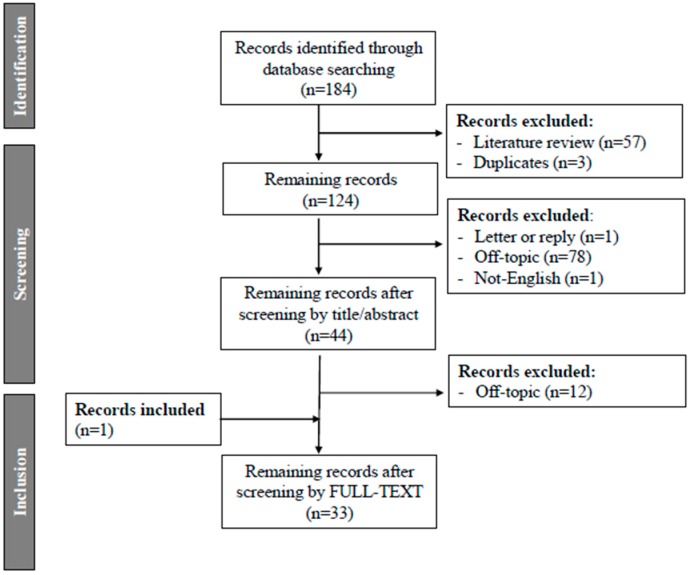
PRISMA flowchart summarizing inclusion of studies in systematic.

No time restrictions were applied. Full-length articles were considered eligible for this systematic review if they were written in the English language. Review articles were excluded during the first screening. Studies found to be irrelevant after reviewing the abstracts were likewise excluded. The remaining articles were retrieved in full-length and assessed according to the eligibility criteria. The reference lists of all known primary articles were examined to identify cited articles not captured by electronic searches. Studies referring to animal models, referring to serum levels of VD metabolites and other endometrial diseases were excluded. Papers without available molecular data about VD pathways in endometrial cells were excluded. Titles and abstracts of all identified studies were screened and the full paper of the preselected articles was read by two researchers (A.M.S., G.C.C.). (Figure 1).

## 3. Vitamin D and Normal Endometrium

During the normal human menstrual cycle, the endometrium undergoes proliferation, differentiation, and finally degeneration. All these events are regulated by changes in steroid hormone levels, mainly estrogen and progesterone. VD, as a steroid hormone, may have a direct role during the modifications that the endometrium undergoes throughout the menstrual cycle [11].

Vienonen and colleagues (2004) were the first to demonstrate the presence of the VDR in human endometrial tissue. They studied the expression pattern of different nuclear transcription factors in normal endometrium including VDR using real-time PCR (RT-qPCR). They evaluated the mRNA expression in uterine samples from three premenopausal women who had undergone hysterectomy (range age 38–50 years). Mainly, they found notable differences in expression levels among individuals, but the levels did not differ between proliferative and secretory phases of the cycle [27]. In 2006, Viganò and colleagues also studied the expression of VDR in the normal endometrium [28]. In that publication, human endometrial samples were collected from women younger than 40 years old, who had not received hormones for at least 3 months and the presence of VDR was demonstrated by RT-qPCR analysis; unfortunately no quantification through the menstrual cycle phases was performed [28]. The group of Linda Giudice conducted a similar study in 2012. They compared the expression of different nuclear receptors in different phases of the endometrial cycle and found that VDR was downregulated in mid secretory phase compared to early secretory phase [29]. Later on, Bergadà and colleagues found different results. In this case, tissue samples in different phases of the endometrial cycle were obtained from 60 women (age ranged from 25 to 55, mean = 43, 20 samples in proliferative and 40 samples in secretory phase), samples were embedded in paraffin blocks and tissue microarrays were analyzed. A decrease in the total expression of VDR in the proliferative endometrium compared with the secretory phase (fold change 3.17, *p* = 0.00002) as well as an increase in the cytosolic VDR protein expression (fold change 1.81, *p* = 0.006) [30] were observed.

The controversial results found in the literature may be explained by the different models and techniques used. Some studies used the cells isolated from tissue that could be contaminated with immune cells present in the endometrium [30] while others used immunohistochemistry analysis of embedded tissue [31]. The selection of the control patients may be as well a source of variability in the different studies. 

The endometrial tissue also expresses the enzymes involved in the metabolism of 1,25(OH)_2_D_3_. The mitochondrial enzyme 1α-hydroxylase, encoded by the *CYP27B1* gene, is expressed in the endometrium [28,31]. Moreover, an increase of enzyme expression has been reported during pregnancy, resulting in higher VD serum level necessary to meet the enhanced calcium requirements during this condition [24]. As previously mentioned, the mitochondrial and microsomal 25-hydroxylases (encoded by *CYP27A1* and *CYP2R1*, respectively), typically present in the liver, were found additionally in the human endometrium [30], where their expression is temporal and, specifically, higher in the secretory compared to proliferative phase of the cycle [30]. 

One of the main functions of the endometrium is to allow for the establishment of pregnancy and changes in human endometrium are essential in this process. Decidualization is the process whereby endometrial stromal cells transform into specialized secretory decidual cells that provide a nutritive and immunoprivileged matrix essential for embryo implantation and placental development [32]. *HOXA10* is a well-known molecule involved in the mechanism of implantation, and a decrease of implantation rates has been observed in women with altered *HOXA10* expression [33]. Indeed, *HOXA10* expression reaches a peak during the window of implantation in response to estrogen and progesterone. In addition, *HOXA10* expression has been found to be regulated by 1,25(OH)_2_D_3_ in human endometrial stromal cells. Therefore, the cross talk between sex steroids and VD may converge in the regulation of *HOXA10* [13]. The link between sex steroids and VD during embryo implantation was confirmed by Viganò and colleagues (2006) [28]. They found that the expression of osteopontin, a progesterone-regulated putative adhesion molecule mediating implantation and decidualization [34], was increased in endometrial cells in response to 1,25(OH)_2_D_3_ [28]. Beside this evidence, the analysis of the VD system on a molecular level in the normal cycling endometrium as well as in pathophysiological conditions has received very limited consideration.

Special attention is drawn to the endometrium of women undergoing assisted reproduction technology. The intake of VD has been shown to improve the thickness of the endometrium in polycystic ovarian syndrome (PCOS) women but not the probability of pregnancy. Unfortunately, no molecular mechanism explaining this phenomenon was proposed [35]. Interestingly, while different hormonal pathways such as insulin and thyroid hormone signaling pathways have been shown to be dysregulated in the endometrium from assited reproductive technology (ART) patients, the hormonal stimulation treatment does not seem to change the VD pathway at least in terms of *VDR* expression [36]. Additionally VD insufficiency has been in the interest of IVF researchers for many years and it remains unknown which element—the endometrium or the oocyte—is more affected by VD deficiency. One of the first studies addressing this issue was from Rudick and colleagues (2014). In that study, they considered the relationship between circulating VD levels in in vitro fertilization (IVF) recipients with pregnancy outcomes, using only donated oocytes to avoid the embryo-oocyte bias in their assessment. Live birth rate resulted lower in the VD-deficient recipients compared to VD-repleted recipients [37]. In contrast, another study reported no differences in implantation and pregnancy rates in egg donation IVF cycles between recipient women with normal or insufficient VD levels [38]. Similar results in terms of implantation rate were found in the study of Franasiak and collegues (2015), where in the case of euploid blastocyst transfer, no differences in pregnancy outcomes between groups with various levels of VD could be found [39]. At this stage, there is insufficient evidence to confirm that VD levels could influence the receptivity of the endometrium of women undergoing IVF.

## 4. Vitamin D and Endometriosis

The expression of *VDR* and VD enzymes in endometriotic tissues was demonstrated for the first time by Agic and colleagues in 2007 [40]. Eutopic endometrial tissues were evaluated by immunohistochemistry in patients undergoing laparoscopy during the proliferative or secretory phase. The control tissue was represented by endometrium from women undergoing laparoscopy for other benign gynecological disorders. *VDR* mRNA expression was evaluated by RT-PCR in eutopic endometrium from women with endometriosis (*n* = 13) compared to that of a control group (*n* = 14). A nonsignificant trend towards higher levels of *VDR* mRNA was observed in the endometriosis group (*p* = 0.10). They analyzed separately epithelial and stromal endometrial cells and reported that *VDR* mRNA was significantly higher in epithelial compared to stromal cells isolated from endometrial biopsies from patients with endometriosis (*p* < 0.01) while this difference could not be detected in the endometrium from healthy controls patients. Nonetheless, the expression of epithelial *VDR* mRNA was higher in the endometriosis group compared to the control group and the same applied to the stromal cell expression. The results of transcriptomic analysis were corroborated by western blot analysis. Unfortunately, the authors did not study the *VDR* mRNA expression in the ectopic endometrium of women with endometriosis. The expression levels of the enzymes 24-hydroxylase, 25-hydroxylase, and 1α-hydroxylase were also evaluated and higher levels of 1α-hydroxylase were found in the endometrium of endometriosis patients compared to the control group (*p* = 0.03). It is still pending whether the dysregulated parameters of VD metabolism are constitutively present in patients with endometriosis or are rather the consequence of a secondary response to the local inflammation [40]. In terms of *VDR* expression, similar results were found in the study by Zelenko and colleagues (2007). These authors analyzed proliferative, early secretory, and midsecretory phase eutopic endometrial samples from control women and endometriosis patients by PCR array and did not find any difference in *VDR* expression levels [29].

Whether the metabolism of VD is dysregulated in the eutopic endometrium of women with endometriosis remains to be elucidated. 

Genetic evidence suggest that polymorphisms in the *VDR* gene may be associated with an altered susceptibility to diseases such as cancer and osteoarthritis [41]. Therefore, this possibility has been also considered in women with endometriosis. The study conducted by Vilarino and colleagues did not find any difference in the frequency of several *VDR* polymorphisms (SNPs) studied by restriction fragment length polymorphisms among 132 women with endometriosis-related infertility, 62 women with idiopathic infertility, and 133 controls [42]. Similar results were reported by Szczepańska and colleagues (2015), as they did not demonstrate differences in genotype and allele frequencies of several *VDR* SNPs between 154 women with endometriosis-associated infertility and 347 controls [43].

Considering the established inverse correlation between VD levels and cancer development [40,44] and the fact that endometriosis is a disease with similar features of a malignancy, the potential mechanistic anti-proliferative and anti-inflammatory effects of VD for the treatment of endometriosis have been investigated in recent years. The molecular mechanism by which VD could affect the development of the disease has been studied mainly in in vitro models with human endometriotic stromal cells [45,46]. In the study from Miyashita and colleagues, endometriotic tissue samples were obtained from the cyst wall of the ovarian endometrioma. Ectopic stromal cells were treated with 1,25(OH)_2_D_3_ and the gene expression profile was analyzed. The authors found a reduction in *IL-1β*, *TNF-α*, metalloproteinase (*MMP*)-2, and *MMP-9* mRNA levels. A reduction of the DNA synthesis was also detected but without affecting the levels of apoptosis [45]. Similar results were reported by Delbandi and colleagues the same year. The ectopic stromal cells isolated from the endometrioma were treated with 1,25(OH)_2_D_3_ and this treatment could significantly reduce *IL-1β*- and *TNFα*-induced inflammatory responses, such as prostaglandin activity, *IL-8* and *MMP* mRNA expression. A significantly reduction in terms of cell invasion and proliferation was also reported [46].

Recently, Ingles and colleagues (2017) have further investigated the pathways regulated by VD in endometriosis cells [47]. An endometriosis stromal cell line (ESC22B) derived from peritoneal endometriosis lesions was treated with the supra-physiologic concentration of 0.1 µM 1,25(OH)_2_D_3_ for 24 h. Using Next-Generation Sequencing, 11,627 differentially expressed genes between treated and untreated cells by at least two fold were detected. The most strongly affected pathways were: (a) the axonal guidance pathway involved in neuro-angiogenesis; (b) the RhoDGI signaling pathways involved in actin organization of the cytoskeleton; and (c) the *MMP* inhibition pathway involved in the degradation of the extracellular matrix. The enzyme 24-hydroxylase and *VDR* were both found to be up-regulated while 1α-hydroxylase, responsible for the conversion of 25(OH)D to 1,25(OH)_2_D_3_ was down-regulated [40]. Finally, the expression of 24-hydroxylase was also compared between eutopic endometrium of healthy subjects and endometriotic lesions of patients. The enzyme was up-regulated in endometriosis lesions indicating, according to the authors, an intense VD metabolism in endometriosis tissues [47].

Generally, a regression of the endometriotic implants after VD or *VDR* agonist treatment has been described mainly using animal models [48,49,50] and several mechanisms are postulated to be involved. For this reason, it is quite surprising that the effect of VD has been investigated on very few cellular functions underlying endometriosis development. Very few data refer to the effect on apoptosis, adhesion, and invasion. Moreover, the impact of VD on endometriosis-mediated inflammatory process has been only vaguely considered. There are however some limitations in the in vitro studies described above that need to be taken into consideration. It should be noted that the characterization of the isolated endometriotic cells from endometriomas does not usually receive the necessary attention that would require a first line cell characterization, hence the possibility of contamination by ovarian components or by fibroreactive tissue is high [51]. Therefore, although these studies may be useful to understand whether the VD treatment may influence the development of the disease, information derived from these studies needs to be considered with caution.

Future investigations need to be performed using different models for endometriosis disease, for instance, primate models, to elucidate the real mechanism by which VD and/or *VDR* agonists may exert an “anti-endometriosis” effect. Finally, clinical trials with VD would be helpful to evaluate the possible therapeutic benefit of VD and/or *VDR* agonists in women with endometriosis.

## 5. Vitamin D and Endometrial Cancer

### 5.1. VD/VDR Pathway and VD Metabolism in Endometrial Cancer

*VDR* protein expression and nuclear localization were for the first time established by immunohistochemistry in human endometrial cancer tissue by Yabushita and colleagues (1996). *VDR* proteins were detected in most human endometrial adenocarcinoma tissues studied (14 of 21 samples). Similarly, some immortalized human endometrial cancer cell lines (RL95-2 and Ishikawa lines) but not all of them (AMEC-1 cell line) expressed detectable levels of *VDR* protein [52]. In order to support the presence of *VDR* in endometrial cancer tissues, ten years later, Agic and colleagues showed by RT-PCR that *VDR* mRNA levels were significantly higher in endometrial cancer tissues compared to endometrial tissue from healthy patients (respectively *n* = 5 versus *n* = 14 patients, *p* = 0.03) [40]. On the other hand, a more recent immunostaining analysis of human tissues has found lower levels of nuclear *VDR* protein expression in 137 tumor samples compared with 55 samples from normal endometrium. Nonetheless, cytosolic *VDR* levels remained unvaried [30]. 

Different components of the VD system have been evaluated in endometrial cancer tissue. As described in healthy endometrium, also in the endometrial cancer tissue the enzyme 1α-hydroxylase has been shown to be expressed in a similar amount between healthy and malignant tissue [31]. On the other hand, a reduced activity of VD has been proposed, resulting from a deficit in the local synthesis due to a reduction of the activity of 1α-hydroxylase and to an increase of VD catabolism. In this context, Agic and colleagues showed significantly increased 24-hydroxylase mRNA levels (*p* < 0.05) in endometrial cancer tissue compared to tissues from healthy control patients [40]. The 24-hydroxylase enzyme is responsible for the catabolism of VD and, potentially, its increase may reduce the cellular effects of calcitriol. Confirming the mRNA expression analysis, 24-hydroxylase protein expression was higher in endometrial cancer cells compared to healthy endometrium as evaluated by western blot and this increase correlated with tumor progression [53]. These results are consistent with an association between increasing mRNA levels of 24-hydroxylase and poor prognosis in high-grade tumor progression in other kind of tissues [54,55]. In disagreement with these findings, Bergadà and colleagues (2014) detected lower levels of 24-hydroxylase protein in tumoral endometrium compared to normal one by immunohistochemical analysis; no differences emerged among samples characterized by different stages of pathology [30]. 

Taken together, the presented molecular evidence could suggest an alteration of VD metabolism in this kind of tumor, suggesting VD as a possible target for potential therapeutic treatments. Nevertheless, the inconsistency of results from the published literature revealed that further studies are required. 

### 5.2. Vitamin D Action in Endometrial Cancer

The first study investigating the effects of VD treatment in an in vitro model of endometrial adenocarcinoma cells did not find any alteration in rate of cell proliferation despite of evident VD responsiveness in terms of 24-hydroxylase activity in these cells [56]. Conversely, the subsequent studies reported an anti-proliferative action of VD in different endometrial cancer cell lines. Saunders and colleagues highlighted the inhibitory effect of VD treatment on the growth of endometrial carcinoma cells (RL95-2) for the first time. Unfortunately, these authors did not investigate the presence of *VDR* and the molecular mechanism to account for the observation [57]. Later, Yabushita and colleagues (1996) demonstrated that the growth of RL95-2 cells expressing *VDR* was prevented in a dose-dependent manner by VD treatment (inhibited to 44% following treatment with 50 nM of 1,25(OH)_2_D_3_ for 6 days); on the contrary, the growth of AMEC-1 cells not expressing *VDR* was completely uninhibited by the treatment [52].

Similar studies proposed VD as an anti-proliferative drug in endometrial cancer cell lines, mainly reporting a mechanism of growth arrest [30,58] or apoptosis [59,60]. Calcitriol treatment induced cell cycle arrest in endometrial cancer cells suppressing some regulators of the cell cycle progression such as cyclin D1 and D_3_ and increasing the expression of p27, a well-known cell cycle inhibitor [59]. In addition, other authors reported variations in expression of proteins involved in several molecular mechanisms such as apoptosis (ex. *hiNT2*), rearrangement of chromatin accessibility (ex. *HIST1H1E*) and differentiation (ex. *EIF2AK2*). Calcitriol was shown to have an additive effect when used in combination with progesterone [59].

Additionally, VD was demonstrated to be able to induce a programmed cell death in endometrial cancer cells activating key actors of the intrinsic apoptotic pathway (such as caspase-3 and caspase-9 proteases) and by disrupting the delicate balance between pro-apoptotic factors and pro-survival defense responses (such as *BAX* versus *BCL-xL* and *Bcl2*) [59,60]. Besides its pro-apoptotic action, Kasiappan and colleagues reported a capacity of VD to suppress the cell survival/proliferation stimuli supported by activation of telomerase typically overexpressed in tumor tissues. In Ishikawa endometrial cancer cells, 1,25(OH)_2_D_3_ treatment induced raising levels of mature miR-498 able to promote the degradation of the human telomerase reverse transcriptase (*hTERT*) mRNA and thus preventing the *hTERT*-supported cell survival with a post-transcriptional gene expression regulation mechanism [61]. 

Vitamin D has been also proposed as a cell-differentiation-inducing agent in endometrial cancer cells. Yabushita and colleagues demonstrated that RL95-2 cells expressing *VDR*, after exposure to 1,25(OH)_2_D_3_ for 6 days, expressed high levels of the cytokeratin polypeptide and became columnar with pronounced polarity and formed gland-like structures when cultured in collagen gel [52]. Haselbergerger and colleagues published similar results [62]. The latter demonstrated an up-regulation of genes involved in differentiation pathways such as E-cadherin and lactoferrin. This effect was dependent on *ICB-1*, a gene involved in the differentiation process of endometrial cancer cells [63]. Additionally, the loss of *ICB-1* blocked the inhibitory effect of VD on the process of epithelial to mesenchymal transition (EMT) [62] that together with migration/invasion and angiogenesis is a fundamental phenomenon in tumorigenesis and cancer progression [64]. In terms of inhibition of angiogenesis, chemotaxis, and endometrial tumor cell growth, a link between semaphorin proteins (*SEMA*) and VD has been as well postulated. Nguyed and collegues reported that *SEMA3B* and *SEMA3F* are strongly induced by 1,25(OH)_2_D_3_ in endometrial cancer cells. Lower receptor levels for these proteins were found in endometrial cancer tissue compared to endometrial tissue from control patients. Importantly, the expression of SEMAs further decreased with the tumor progression, suggesting SEMAs as onco-suppressor genes with a key role in molecular mechanisms of transforming processes [58]. More recently, using Ishikawa endometrial cancer cell line, the 1,25(OH)_2_D_3_ treatment was shown to affect the reorganization of the cytoskeleton mainly down-regulating the expression and activity of proteins involved in the reorganization of actin structures (such as Actin-Related Protein *ARP2*, Rac Family Small GTPase *RAC*-*1* and *PAK1* kinase protein) and inducing de-polymerization of actin filaments [65]. Vitamin D may also be a modulator of invasiveness of endometrial tumor cells. Indeed, Bokhari and colleagues found a weak reduction of the invasive potential of endometrial cancer cells (HEC-1B and Ishikawa cells) (15–20%) and a reduction in the levels of tumor invasion molecular markers such as *MMP2* and *MMP9*, upon VD treatment [53]. The inhibitory effect of VD was more effective with a progesterone-induced 24-hydroxylase inhibition in line with the observation that 24-hydroxylase is upregulated in endometrial cancer cells and can control VD cellular responses [53]. This result is consistent with an enhanced anti-proliferative action following a combination of VD and 24-hydroxylase inhibitor treatment in in vitro and in vivo models of prostate and lung tumors [66,67].

Finally, VD is a putative anti-inflammatory agent and inflammation is widely considered as a risk factor for cancer development [68]. In addition, several studies showed a reduction of endometrial cancer risk associated with the use of anti-inflammatory agents [69,70]; therefore, the role of the anti-inflammatory action of VD in endometrial cancer has been closely investigated. VD-induced suppression of NF-κB, the key transcription factor involved in inflammation and innate immunity responses, was associated with a diminished expression of inflammatory cytokines/chemokines involved in metastasis-related processes such as *CXCL1* and *CXCL2* [71].

The results of this review showed the widespread effects of VD of endometrial cancer cells. The complex pleiotropic effect of VD on endometrial cancer cells can be exemplified with the results of the study by Lee and colleagues. These authors revealed that treatment of the HEC-1B cell line with 1,25(OH)_2_D_3_ could modify the expression of more than 300 proteins among which oncogenes, tumor suppressor, membrane and structural proteins and actors in a multiplicity of processes such as cell cycle, transcriptional regulation, differentiation, and DNA repair [59].

Although the evidence is suggestive of an inhibitory effect of VD on endometrial cancer, it is important to underline that the majority of the studies concerning VD and endometrial cancer has been done using immortalized endometrial cancer cells. Moreover, the findings on in vivo or ex vivo studies to investigate molecular mechanisms of VD in endometrial cancer are scarce and therefore warrants further investigation.

## 6. Conclusions

The role of VD and female fertility has been deeply studied. However, the role of VD in physiological endometrium has been less considered. The results of this review demonstrate that the knowledge about the effects of VD in physiological endometrium is poor and that the molecular mechanisms involved are still to be completely defined.

One of the purposes of this review was to elucidate the role of VD in the physiologic endometrium, in endometriosis, and in endometrial cancer. Apparently, *VDR*-mediated signaling pathways seem to be dysregulated in those pathological conditions; nonetheless, the results are contradictory (Table 1). Therefore, more studies are needed to confirm a beneficial role of VD treatment on endometrial cancer and/or endometriosis.

## Figures and Tables

**Table 1:** Studies addressing the presence/absenceof *VDR* and enzymes that metabolized VD in normal and pathologic endometrium.

Type of Samples	Target	Result	Technique	Reference
Endometrial tissue from control patients (premenopausal)	*VDR*	Presence	PCR-array	Vienonen et al., 2004 [27]
		Presence		Vigano et al., 2006 [28]
		Down-regulated in mid-secretory vs. early secretory	PCR-array	Zelenko et al., 2012 [29]
		Down-regulated in proliferative vs. secretory phase	Tissue array	Bergada et al., 2014 [30]
	1α-hydroxylase	Presence	RT-PCR	Vigano et al., 2006 [28]
		Presence		Becker et al., 2007 [31]
		Down-regulated in proliferative vs. secretory phase	Tissue array	Bergada et al., 2014 [30]
	25-hydroxylase	Down-regulated in proliferative vs. secretory phase	Tissue array	Bergada et al., 2014 [30]
Eutopic endometrium from endometriosis patients	24-hydroxylase	Up-regulated endometriosis vs. control tissue	RT-PCR	Agic et al., 2007 [40]
	25-hydroxylase	No differences between endometriosis vs. control tissue		
	1α-hydroxylase	No differences between endometriosis vs. control tissue		
	*VDR*	No differences between endometriosis vs. control tissue		
		No differences between endometriosis vs. control tissue	RT-PCR	Zelenko et al., 2012 [29]
Endometrial tissue from endometrial cancer patients	*VDR*	Up-regulated endometrial cancer vs. control tissue	RT-PCR	Agic et al., 2007 [40]
	Nuclear *VDR*	Down-regulated endometrial cancer vs. control tissue	Tissue array	Bergada et al., 2014 [30]
	24-hydroxylase	Up-regulated endometrial cancer vs. control tissue Correlation with tumor progression.	Tissue array	Bokhari et al., 2016 [53]
		Down-regulated endometrial cancer vs. control tissue No differences in tumor progression.		Bergada et al., 2014 [30]
		Up-regulated endometrial cancer vs. control tissue	RT-PCR	Agic et al., 2007 [40]
	1α-hydroxylase	No differences between endometrial cancer vs. control tissue	Immunostaining	Becker et al., 2007 [31]
